# A Promising Method for the Determination of Cell Viability: The Membrane Potential Cell Viability Assay

**DOI:** 10.3390/cells11152314

**Published:** 2022-07-27

**Authors:** Eneko Madorran, Andraž Stožer, Zoran Arsov, Uroš Maver, Jan Rožanc

**Affiliations:** 1 Institute of Anatomy, Faculty of Medicine, University of Maribor, Taborska Ulica 8, 2000 Maribor, Slovenia; 2 Institute of Biomedical Sciences, Faculty of Medicine, University of Maribor, Taborska Ulica 8, 2000 Maribor, Slovenia; uros.maver@um.si; 3 Department of Pharmacology, Faculty of Medicine, University of Maribor, Taborska Ulica 8, 2000 Maribor, Slovenia; 4 Institute of Physiology, Faculty of Medicine, University of Maribor, Taborska Ulica 8, 2000 Maribor, Slovenia; andraz.stozer@um.si; 5 Department of Condensed Matter Physics, Jozef Stefan Institute, Jamova 39, 1000 Ljubljana, Slovenia; zarsov@biosistemika.com; 6 BioSistemika d.o.o., Koprska Ulica 98, 1000 Ljubljana, Slovenia; 7 BioCore Institute, Nad Izviri 8, 2204 Miklavz na Dravskem Polju, Slovenia

**Keywords:** cell viability, cell culture, membrane potential, cell death

## Abstract

Determining the viability of cells is fraught with many uncertainties. It is often difficult to determine whether a cell is still alive, approaching the point of no return, or dead. Today, there are many methods for determining cell viability. Most rely on an indirect determination of cell death (metabolism, molecular transport, and leakage, to name a few). In contrast, we have developed a promising novel method for a “direct” determination of cell viability. The potential method assesses cell membrane integrity (which is essential for all viable cells) by measuring the electrical potential of the cell membrane. To test the assay, we chose two different cell types, blood macrophages (TLT) and breast cancer epithelial cells (MCF 7). We exposed them to seven different toxic scenarios (arsenic (V), UV light, hydrogen peroxide, nutrient starvation, Tetrabromobisphenol A, fatty acids, and 5-fluorouracil) to induce different cell death pathways. Under controlled test conditions, the assay showed good accuracy when comparing the toxicity assessment with well-established methods. Moreover, the method showed compatibility with live cell imaging. Although we know that further studies are needed to confirm the performance of the assay in other situations, the results obtained are promising for their wider application in the future.

## 1. Introduction

Cell viability is a key parameter for any cell-based model, especially toxicological cell-based models. For this reason, many assays have been developed to determine cell viability. Usually, these assays are based on the determination of metabolic activity, plasma membrane integrity, apoptotic markers, mitochondrial markers, and “targeted” proliferation assays [1,2]. Despite their undeniable utility for cell analysis, these methods are prone to false-negative and false-positive errors. These errors occur, for example, when cell metabolism changes, when the cell death pathway changes, or when a molecular conformational change occurs (which may even be independent of the cell death pathway), to name a few examples [1,2,3]. In addition, we must consider a large number of processes that occur simultaneously in a viable cell and that can affect the performance of the assay [4]. Cell death is only one of these processes (independent of the many steps from which it results) that is definitive. Therefore, measuring cell death should not be as complex as determining cell viability, although it provides the same information (considering that life and death are exhaustive terms). However, the determination of cell death is still subject to certain limitations, most of which are related to problems of the reversibility of the determination. In this sense, any reliable method for determining cell death should reveal a specific process within the cell death cascade that occurs after the point of no return. To overcome this, we have developed a new measurement approach that focuses on the permanent loss of cell membrane barrier function, which is essential for all viable cells [2]. The permanent loss of membrane integrity leads to an irreversible increase in resting membrane potential, i.e., depolarization, due to a loss of selective permeability, ion concentration gradients, and mass electroneutrality [5,6]. Certain cells can increase their membrane potential for a short time and then decrease back to their resting membrane potential. Nevertheless, a cell that increases its membrane potential for a certain time (and does not decrease back) has most likely lost its membrane integrity and can be considered dead [2]. Consequently, we can determine the viability of the cell by measuring the membrane potential.

A previous study indicated the possible use of membrane potential as a marker of cell viability. In this study, the authors established a relationship between the contribution of ion gradients (mainly sodium and potassium) to apoptosis, and the subsequent loss of cell volume and change in cell membrane potential [5]. The authors also emphasized the role of monovalent ions in apoptosis. However, they did not focus on membrane potential changes as a marker of cell death, which is the focus of our study. We developed an assay based on the measurement of the cell membrane electrical potential, the Membrane Potential Cell Viability Assay (MPCVA).

## 2. Materials and Methods

### 2.1. Chemicals

The chemical 3-(4,5-dimethylthiazole-2-yl)-2,5-phenyl tetrazolium bromide (MTT) was acquired from Sigma-Aldrich (Saint Louis, MO, USA). Phosphate-buffered saline solution (PBS), FluoVolt™ (FV) Membrane Potential Kit, Vybrant™ DyeCycle™ Ruby Stain (Vybrant), and propidium iodide (PI) were purchased from Thermo Fisher Scientific (Waltham, MA, USA).

Sodium arsenate dibasic heptahydrate was purchased from Sigma (Roedermark, Germany) to induce cell death. The chemical was mixed with the cell culture medium to achieve a final concentration of 1 mg arsenic (V)/L medium and 5 mg arsenic (V)/L medium. These concentrations showed cytotoxic effects in previous studies under similar conditions [7,8,9,10].

Hydrogen peroxide (30%), Tetrabromobisphenol A (TBBPA), 5-Fluorouracil, and Triton X-100 were purchased from (Merck KGaA, Darmstadt, Germany). Fatty acids were obtained from oil digestion with lipase (Merck KGaA, Germany).

### 2.2. Cell Culture

MCF-7 cells were purchased from ATCC (Manassas, VA, USA). Human blood macrophages, called TLT, were isolated from the blood of a healthy donor at the University of Maribor (Maribor, Slovenia) [10]. TLT and MCF7 cells were cultured in colorless Williams E medium (Thermo Fisher Scientific, Waltham, MA, USA) supplemented with 5 wt% and 10 wt% fetal bovine serum (Gibco, Thermo Fisher Scientific, Waltham, MA, USA), respectively. L-glutamine (2 mM, Sigma), penicillin (100 U mL^−1^, Sigma), and streptomycin (1 mg∗mL^−1^, Fluka, Buchs, Switzerland) were also added. Cells were cultured in 25 cm^2^ culture flasks (Corning, New York, NY, USA) at 37 °C and 5% CO_2_ atmosphere by weight until the desired degree of confluence (50, 75, or 100%) was achieved.

To observe possible differences in the performance of the assay depending on the cell origin, two different cell types (macrophages and epithelial cells) were used in our experiments. One of these two cell types was a primary cell and the other was a carcinogenic cell line, so that we could compare the different cell death pathways. Considering that carcinogenic cells have a disrupted cell death pathway, we observed different trends of cell death.

### 2.3. The Mechanism behind the Hypothesis

Cell membrane potential, by definition, is the electrical potential difference between the inside and the outside of the cell. The potential difference is maintained as long as the cell maintains its membrane integrity. When the cell initiates the death pathway, the integrity of the cell membrane is compromised and can abolish the electric potential difference between the cell interior and exterior, due to bulk mass electroneutrality (0 mV) [5,6,11,12,13]. Therefore, our proposed membrane potential cell viability assay can be used to determine this membrane potential change, and thus, cell viability.

**Figure 1:**
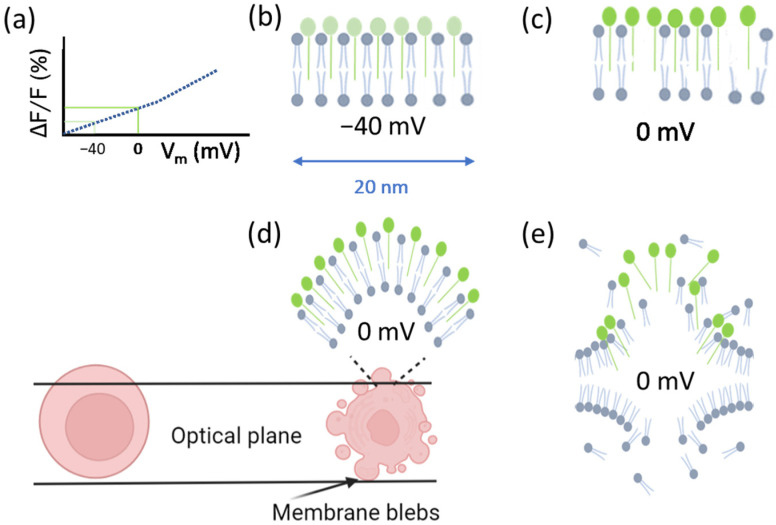
Dye dynamics in relation to cell viability. (**a**) Dye fluorescence intensity is proportional to membrane potential. (**b**) A cell’s resting membrane potential is negative. (**c**) Depolarization of cells due to loss of membrane integrity increases dye intensity. (**d**) Depolarization of cells also occurs during apoptosis, which increases dye intensity. Additionally, formation of apoptotic bodies increases the surface area of the membrane, thus increasing the number of dye molecules in a given optical plane. (**e**) When the cell membrane loses its integrity to the stage of membrane disintegration, the dye molecules detach. This significantly decreases the dye intensity due to the reduction in the number of dye molecules (despite a possible concomitant depolarization of the cell).

Some dyes allow for the measurement of cell membrane potential via the correlation with fluorescence intensity [14,15]. An example is the commercially available Fluovolt™ dye (FV), whose intensity rapidly responds (25%/100 mV) to changes in membrane potential (Figure 1a). The dye consists of three elements: the fluorophore (which is aligned with the outer layer of the membrane and has low internalization), the wire (which has low attenuation), and the donor (which has high voltage sensitivity). The voltage sensitivity of the donor is related to the local electric field, which affects the free energy for photoinduced electron transfer (PeT). PeT occurs from the donor through the wire to the fluorophore, and at lower membrane potentials (e.g., normal or resting membrane potential values) PeT is more favorable, while at higher membrane potentials it is less favorable. Therefore, a depolarization of the cell (for instance from −70 mV to 0 mV) should induce higher fluorescence emission, since PeT and fluorescence are competing processes [16]. With this in mind, a viable cell with a negative resting potential [12,17] (Figure 1b) that loses its membrane integrity should subsequently depolarize. Additionally, this depolarization should lead to an increase in dye intensity (Figure 1c). When the cell follows apoptotic cell death and forms apoptotic bodies, the surface area of the cell membrane [18] increases due to the formation of blebs, and so does the number of dye molecules contained in the optical plane, which should further increase the total dye intensity and account for increases in fluorescence above the increases due to depolarization (Figure 1d). When the cell membrane damage reaches the stage of membrane disintegration, the dye intensity decreases significantly, due to a loss of dye molecules (despite the fluorescence increase due to the depolarization of the cell) (Figure 1e).

A previous study in which the authors used FV to measure the cell membrane potential in HEK (kidney) cells [19] suggested the suitability of the dye for measuring membrane potential in cells. Another study showed that increasing the external potassium (K^+^) concentration to 15, 30, and 40 mM induces membrane depolarization of rat neurons to −56, −26, and −19 mV, respectively [20]. To test the suitability of the dye FV for the selected cell lines (MCF7 and TLT), cells were loaded with FV and cultured in cell culture media with different K^+^ concentrations to confirm that the intensity of FV increases with an increase in membrane potential. Initially, cells were cultured in a 5 mM medium (as described in Appendix A) for 30 min. After adjusting the cells to the new medium, the cell culture medium was modified by gradually increasing the K^+^ concentration to 15 mM, 30 mM, and finally 50 mM. Cells were observed using the FITC/GFP fluorescence and brightfield channels of a LEICA DMI 6000B fluorescence microscope (see “Section 3.1. Suitability of Fluovolt for Measuring Membrane Potential in MCF7 and TLT” for more information).

After testing for dye suitability, MCF7 and TLT cells were treated (allowed to die) with 0.20 mM Triton X-100, to observe differences between the dye intensities in treated and untreated cells [21,22]. The measurement was performed using an image flow cytometer, Image stream MK2 (ISX) (Luminex, Austin, TX, USA).

Considering the properties of the dye and the fact that cell death always leads to membrane rupture, we expect a low incidence of false-negative results when testing cell viability with the MPCVA assay. On the other hand, a transient loss of membrane stability that does not result in cell death could lead to a higher rate of false-positive results with this assay (a common cause of false-positive results with the PI dye). As we note several times in the manuscript, duplicates should be measured with a difference of one hour. Most likely, such a long loss of membrane potential is not reversible, and is accompanied by cell death.

### 2.4. Fluovolt Dye Measurement with a Fluorescence Microscope

**Figure 2:**
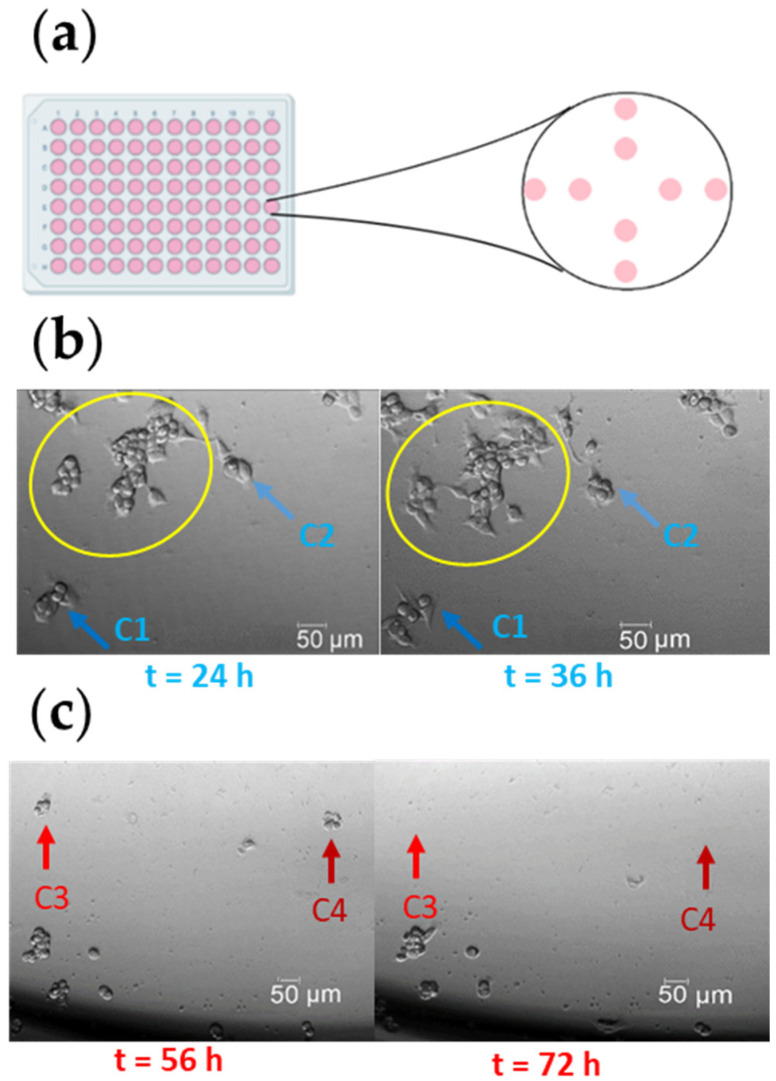
The experimental approach to classify viable or dead cells. (**a**) Schematic representation of cell seeding. (**b**) The clusters of cells marked with a yellow circle were excluded from the analysis. Cells marked with a blue arrow (dark blue arrow, C1; or light blue arrow, C2) were recorded at t = 24 h and t = 36 h for comparative analysis, to identify proliferating cells. These cells were classified as viable cells. (**c**) Cells marked with red arrows (light red arrow, C3; or dark red arrow, C4) were recorded at t = 56 h and t = 72 h for comparative analysis, to identify lysed cells. These cells were classified as dead cells.

Small cell colonies (up to 100 cells) were seeded in 96-well plates at different locations in the wells, and allowed to adhere overnight (Figure 2a). After 24 h, all cells were stained with FV according to the protocol described in Appendix, Protocol A1. Arsenic (1 mg/L) was added to randomly selected wells as a “killing agent” to induce cell death [9,10]. Cells were observed for a period of up to 80 h, and categorized according to their viability (without considering whether or not the cells were cultured with arsenic). We defined viable cells as those that replicated (Figure 2b, blue arrows) and cells that lysed as dead cells (Figure 2c, red arrows). Only small cell colonies were included in the experiment, to track individual cells. Larger cell colonies were excluded, to avoid errors in identifying cell replication or cell death (Figure 2b, yellow circle).

**Figure 3:**
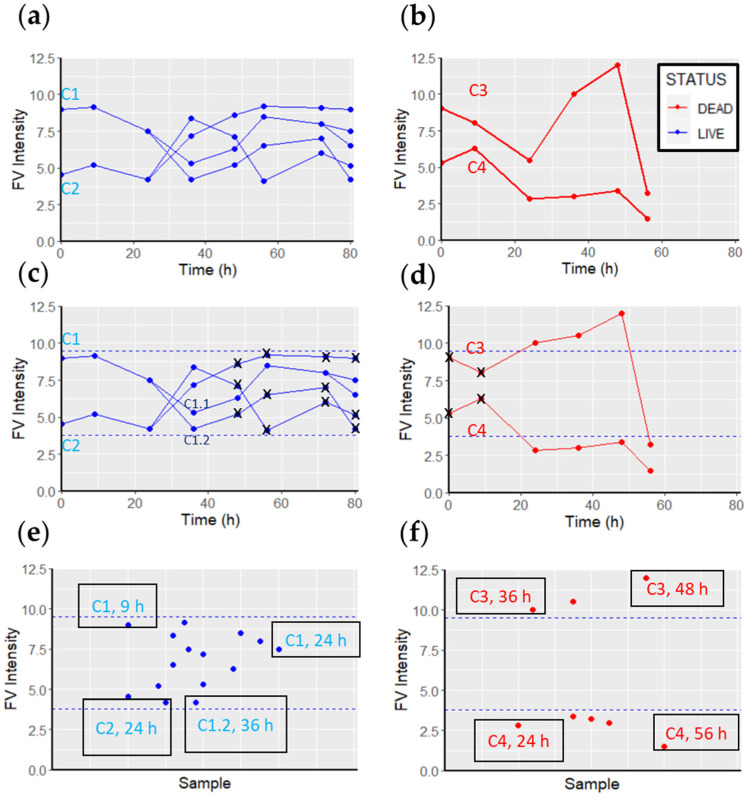
Correlation between FV fluorescence intensity, and cell viability or death. (**a**) FV intensity values measured in viable cells. (**b**) FV intensity values measured in dead cells. (**c**) Cell C1 replicated at t = 36 h, and its daughter cell, C1.1, at 80 h. The FV intensity values of the C1 cell for up to 36 h were recorded as viable FV intensity values. Similarly, all FV intensity values of the C1.1 cell up to 80 h were accounted for as viable FV intensities. The C1.2 cell did not replicate, so its FV intensity values were discarded (black crosses). Cell C2 replicated at t = 36 h. After this replication, none of the daughter cells replicated. Therefore, all FV intensity values up to 36 h are accounted for as viable FV intensity values, and the rest are discarded (black crosses). All viable FV intensities are considered to form the viable FV intensity range (within the dashed blue lines) (15 values in this example). (**d**) Both cells (C3 and C4) were lysed between the 56 and 72 h measurements. Therefore, the measurements at t = 0 h and t = 9 h are discarded (black crosses), as they are not within the 48 h span. (**e**) All viable intensities of FV recorded during the 80 h monitoring period constitute the “live cell sample” (15 values in the example). (**f**) All intensities of FV recorded during the 80 h monitoring period that are associated with dead cells constitute the “dead cell sample”.

The intensity of FV was measured for each selected cell every 12 h with an automatically controlled fluorescence microscope until the end of the experiment (or cell lysis), and related to their viability. Cells that replicated during monitoring were classified as viable (Figure 3a), and all previously measured FV fluorescence values until cell division were assigned to viable FV intensities. The mean of all FV intensities was calculated, and a range was determined by considering two standard deviations from the mean. Therefore, values outside these two thresholds were not considered viable FV intensity values (Figure 3c) (these ranges are commonly used in biochemical assays in clinical diagnostics). Such extreme values may occur because of a brief depolarization caused by a change in metabolism or a transient disruption of the cell membrane (e.g., exocytosis, endocytosis, or pinocytosis). These potential artifacts occur sporadically and were therefore rare (thus, only the most extreme values were discarded). Nevertheless, the determination of the range may be modified depending on the needs of the experiment (e.g., due to the specifics of each cell type to reduce potential false positives or false negatives).

Similarly, fluorescence was measured in cells undergoing lysis during monitoring (Figure 3b). Considering that the initiation of the cell death process can take between 2 and 48 h [23,24,25,26,27], it is difficult to know when exactly a cell initiates the cell death pathway, and when it crosses the point of no return. With this in mind, we classified the FV intensity values of a cell within 48 h before cell lysis as “dead cell” FV intensity values (knowing that it is possible for cells to initiate the cell death pathway later than 48 h before lysis) (Figure 3d). The FV intensity values in viable cells (Figure 3e) were statistically compared with the FV intensity values in dead cells (Figure 3f) to detect significant differences between them. To minimize possible external stress on the cells, we performed the measurement as quickly as possible, usually in less than 10 min. A total of N = 164 MCF7 and N = 151 TLT cells were analyzed. The FV intensity values of each cell at each recording time point were normalized to background intensity [28] in Equation (1):

(1) $$\frac{FV\;intensity\;of\;the\;cell}{Background\;FV\;intensity\;of\;correspondent\;ROI\;},$$

### 2.5. Assessment of Cell Viability with a Multispectral Imaging Flow Cytometer Amnis Image Stream Mark II

**Figure 4:**
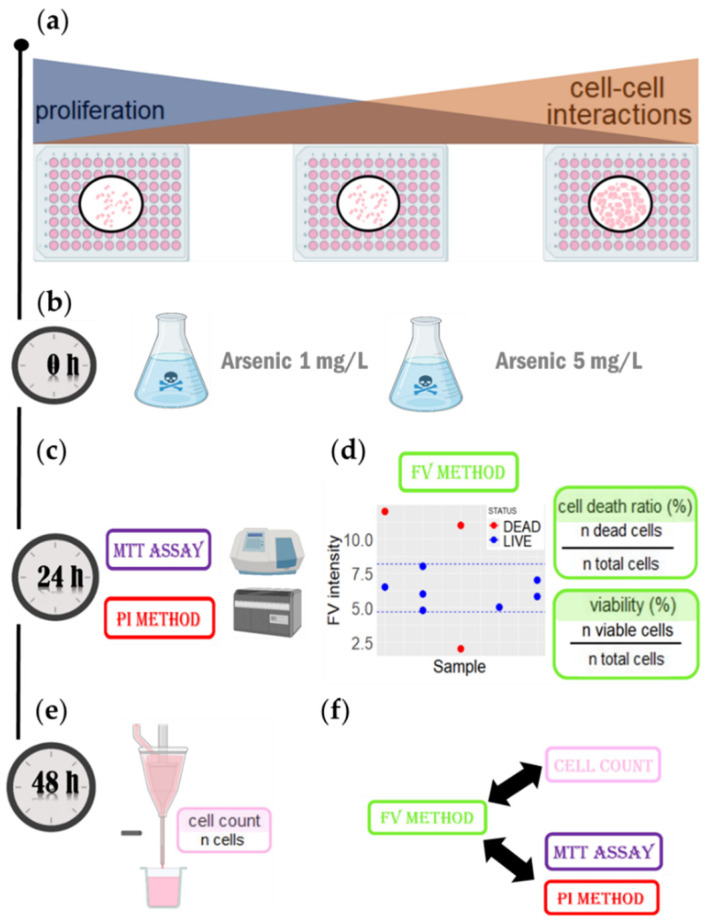
The workflow to evaluate MPCVA accuracy. (**a**) Cells were seeded at different concentrations to achieve different confluency (50%, 75%, and 100%, respectively). (**b**) After the cells were seeded, arsenic (V) (1 mg/L and 5 mg/L) was added to the treated groups. (**c**) At 24 h after treatment, cell viability was determined using MTT assay and PI method. (**d**) At the same time, the MPCVA method was performed. In the side scatter plot, the dashed blue line represents the FV viable intensity range, based on which cell viability and cell death were calculated. (**e**) At 48 h after treatment, cell viability was determined using the cell count method. (**f**) The accuracy of the MPCVA method was compared with the cell count method, as well as with the MTT assay and the PI method.

Following the standard protocol for assessing cell viability [29], cells were seeded in 96-well plates with different cell concentrations to achieve different levels of confluency, namely 50% (CONF50), 75% (CONF75), and 100% (CONF100), respectively (Figure 4a). At a lower confluency, cell replication was abundant and there were fewer cell–cell interactions. When increasing the confluency, both parameters change inversely. When cells were attached (24 h after the seeding), they were treated with arsenic at two different concentrations (1 mg/L and 5 mg/L) for 24 h (Figure 4b). After treatment, cells viability was examined using four different cell viability assays: the MPCVA assay (Figure 4d), propidium iodide (PI) [30], the 3-(4,5-dimethylthiazol-2-yl)-2,5-diphenyltetrazolium bromide (MTT) assay [31] (Figure 4c), and cell counting using a flow cytometer [32]). (Figure 4e). The latter method had the lowest number of false-negative viability events because dead cells are determined as cells that are not counted (if the cell lyses, it is certainly dead). False-positive events should also have been limited, since a cell that has not lysed 48 h after treatment should be viable (at that point). Furthermore, a cell that has passed the point of no return in its cell death pathway (which is what the MPCVA measures) will most likely be lysed in the following 24 h.

In the MPCVA method, all experiments were performed in duplicate. Duplicates of each group were measured with a difference of 1 h to ensure that the difference in cell membrane potential was due to permanent (not temporal) membrane depolarization. The final FV intensity values were compensated for by the following formula (FV intensity normalized to the FV dye area) to minimize/exclude the influence of differences in cell size or dye penetration in the cells on the overall FV fluorescence intensity value in Equation (2):

(2) $$\frac{Total\;FV\;intensity}{The\;area\;covered\;by\;the\;FV\;dye},$$

After compensation of the FV intensity values, the range of viable FV intensity was delimited by the FV intensity values of the untreated cells that were within two standard deviations from the mean. Thus, viable cells were determined as cells whose FV intensities were within the viable FV intensity range, whereas cells whose FV intensities were above or below this range were considered dead. From these perspectives, the cell death ratio and cell viability were calculated as described in Figure 4d.

Fluorescence intensity and cell number were analyzed using the Amnis Image Stream Mark II (ISX) multispectral imaging flow cytometer (Amnis, Seattle, WA, USA). The absorbance of the samples in the MTT assay were measured using VARIOSKAN spectrophotometer (Thermo Scientific, Waltham, MA, USA).

### 2.6. Evaluation of the Method Using Alternative Cell Death Pathways and Comparison with Standard Methods

The suitability of the method for use in alternative cell death situations (i.e., exposure of cells to various chemical and physical agents) was another important goal in its evaluation. First of all, it must be acknowledged that it is quite difficult to induce a specific cell death pathway, since the same toxic agent may induce different cell death pathways, depending on the experimental conditions [33,34,35]. Moreover, a comprehensive study of cell death pathways will also reveal new pathways. With this in mind, we decided to test the suitability of the assay on blood macrophages, as they can undergo different cell death pathways (apoptosis, necroptosis, ferroptosis, parthanatos, etc.) [36]. Although we did not investigate the possible correlation of the test results with a specific cell death pathway, we still believe that we covered different cell death pathways using different cell death inducers with the proposed experimental setup.

Blood macrophages were grown to confluence in 96-well plates when the treatment was added. The following chemicals were selected from the various toxic substances found in the literature: H_2_O_2_, TBBPA, and fluorouracil. It was expected that 12.5 mM H_2_O_2_ would induce necroptosis (also known as parthanatos), while 266 µM TBBPA and 500 µM fluorouracil were expected to induce apoptosis [37,38,39]. Cell death was also induced by nutrient starvation (mainly autophagy) [40]. For this purpose, 50 microliters of the cell medium was added, and the medium was not replenished for 72 h. To induce ferroptosis (via lipid peroxidation) [33], 15 µL of fatty acid was added to the cell medium. Finally, cell death (mainly parthanatos and apoptosis) was also induced by exposing cells to UV radiation for 5 min [41]. All concentrations and exposure times were optimized based on the preliminary experiments (Figure S1).

### 2.7. Short Description of Standard Viability Methods

#### 2.7.1. Cell Count

Cell density and treatment before dye staining were determined by the experimental setup. The cell number in each sample was measured 48 h after treatment with ISX (Channel IX, brightfield). Considering that each cell death pathway usually does not last longer than 48 h [23,24,25,26,27], cells that initiate the cell death process after inoculation of the toxicant should be lysed before 48 h. The cell number of each sample was determined by counting 2000–5000 cells from a limited volume of the sample (10–30 µL). The final cell number was determined by considering the concentration measured with the ISX and the total volume of the sample (50 µL). Under these conditions, this method was considered the most accurate (and at the same time, the most time-consuming, at 48 h) method to evaluate the viability of the cells.

#### 2.7.2. Propidium Iodide Method

Cell density and treatment before dye staining were determined by the experimental setup. Cells were stained with 50 µg/mL propidium iodide 24 h after treatment to determine cell viability with respect to dye penetration. The presence of the dye in the cell was determined with the ISX by exciting the dye with a laser at a wavelength of 488 nm, and observing the emission in the IV channel. The cell density and treatment were determined by the experimental setup.

#### 2.7.3. MTT Assay

The cell density and treatment before dye staining were determined by the experimental setup. Cellular metabolic activity was measured with 3-(4,5-dimethylthiazol-2-yl)-2,5-phenyltetrazolium bromide (MTT; Sigma) to determine cell viability 24 h after treatment. Cells were stained with a final concentration of 0.5 mg/mL and incubated for 2 to 4 h. The absorbance of the converted dye was measured using VARIOSKAN at a wavelength of 570 nm.

### 2.8. Statistical Analysis

When analyzing the differences between live and dead cell groups using the fluorescence microscope (“Section 3.2. The behavior of cells stained with Fluovolt in relation to cell viability”), the Mann–Whitney test was used. This test was chosen because of the FV intensity distribution of the dead cells (high and low values can be classified with this test). The Chi-squared test was used to evaluate the performance of MPCVA in “Section 3.3. Performance of predicting cell viability of FV-stained cells with ISX”. All statistical analyses in this study were performed using the R program.

## 3. Results

### 3.1. Fluovolt (FV) Suitability for Measuring Membrane Potential

**Figure 5:**
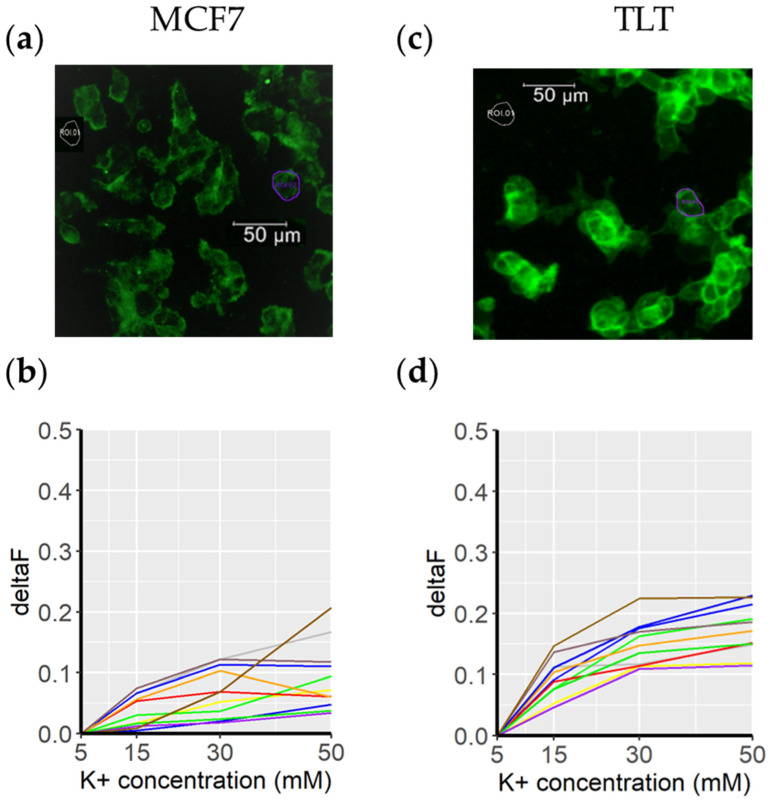
Fluovolt dye intensity in response to a depolarizing medium. (**a**) A representative image of MCF7 cells acquired with the FITC/GFP channel. The purple circle (ROI2) marks the cell in which the membrane potential was measured (for clarity, the other cells are not circled). The background signal is shown in gray and is labeled ROI1. The membrane potentials of the cells in this image are shown below, in (**b**) (each colored line represents a cell from the top image). Cells were cultured with cell culture medium in which the K+ concentration was gradually increased (from 5 mM to 50 mM. The same procedure was applied to the TLT cell line (subfigures (**c**) and (**d**)).

The first part of this study was to confirm the suitability of FV as a membrane potential marker. Target cells were cultured with a medium with gradual changes in K^+^ concentration (5 mM, 15 mM, 30 mM, and 50 mM), taking into account the changes in membrane potential observed in previous studies [20]. Analyzing the FV intensity values of individual cells via the gradual change of K^+^ in the medium, the intensity change of FV implies proportionality with the membrane potential (Figure 5b,d). Consequently, the change in cell membrane potential is measurable with the FV dye in both cell lines tested, MCF7 (breast cancer) and TLT (macrophages), and the change in the fluorescence intensity of FV is proportional to the change in membrane potential. The magnitude of the change in fluorescence intensity is consistent with the specifications of the dye [16].

Both cell lines showed similar resting membrane potentials (around –40 mV) [42,43] and responded similarly to changes in K+ concentration. Higher intensity variations are expected when analyzing cell viability, as the membrane potential continues to increase when the cells die (0 mV). Furthermore, given the considerations explained in Figure 1 (morphological changes affecting dye concentration in a given surface area/optical plane), larger FV intensity variations can be expected for observations on dying cells.

When assessing the toxicity of the dye, comparable cell proliferation was observed between stained and unstained cells. Thus, the dye is evaluated as being non-toxic, which is consistent with several previous studies [13,18,43].

### 3.2. The Behavior of FV When Culturing Cells with Triton

**Figure 6:**
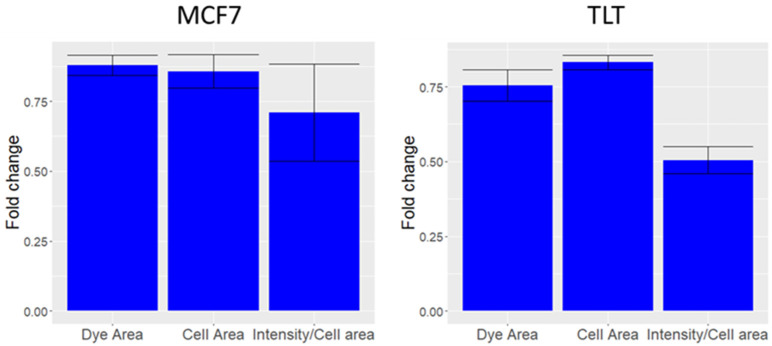
The effect of Triton x20 on MCF7 and TLT cells. (**MCF7**) The change in area covered by the dye (Dye area), area of the cell (Cell area), and compensated intensity (total FV intensity/Dye Area) of MCF7 cells treated with Triton^TM^ X-100 with respect to untreated MCF7 cells. (**TLT**) The same procedure was applied to the TLT cell line.

Triton X-100 washes off the cell membrane (measured as a reduction in cell area in Figure 6), which can subsequently lead to the leakage of the dye from the cell membrane. This in turn causes a reduction in the intensity of FV, despite the depolarization of the cells. The reduction in dye intensity is much greater than the reduction in area. However, it should be remembered that ISX (and most flow cytometers) are not capable of measuring localized cell membrane losses [44]. Therefore, we assume that the difference in compensated FV intensity is due to Triton-induced cell membrane disintegration (since Triton X-100 is known to disintegrate the membrane [21]). The data showed in Figure 6 confirms our assumption presented in Figure 1e; because Triton X-100 causes significant membrane damage in a short period, we expect cells that are undergoing lysis to have similar dynamics and lower FV intensity values than live cells.

### 3.3. The Behavior of FV-Stained Cells When Evaluating Cell Viability

**Figure 7:**
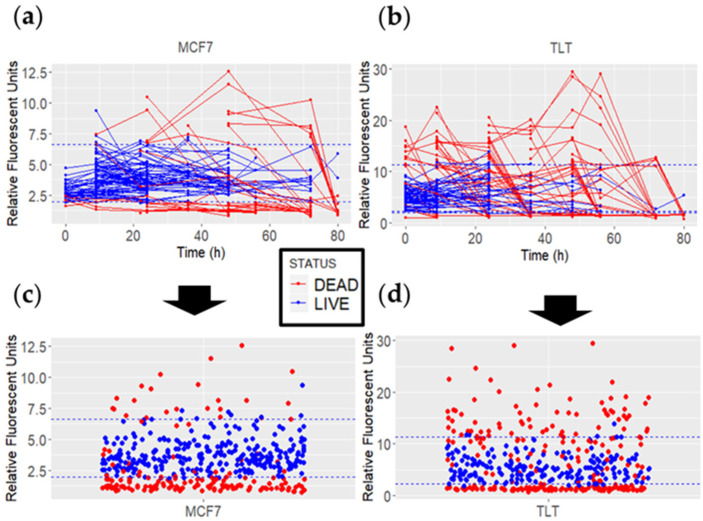
MCF7 and TLT FV fluorescence intensity values of dead (red) and viable (blue) cells over 80 h. The dashed blue line represents the viable FV intensity range. (**a**,**b**) The lines connect the FV intensity values of each cell over time. (**c**,**d**) All measured data points for each cell line are plotted as individual points.

Measuring the FV intensity values of individual cells throughout the experiment (Figure 7), we found that live cells (shown in blue) did not exhibit sudden FV intensity changes. Furthermore, the FV intensity values were similar between the different viable cells. In contrast, two main trends were observed in dead cells: In the first trend, the FV intensities of the cells slowly decreased until they were lysed. In the second trend, the FV intensity values increased significantly and then decreased (Figure 7b). However, with the information obtained, we could not precisely relate the trends to specific cell death pathways. At this stage, the most valuable information was that the viable cells had similar FV intensity values that were different from the “dying” cells. A statistical analysis of FV fluorescence intensity between the dead and viable cells confirmed that the difference was significant (MCF7; W = 35,106, *p*-value < 2.2 × 10^−16^. TLT; W = 27,176, *p*-value = 0.03164).

Some false-negative results (red dots within the viable FV intensity range) were observed when analyzing the data. Such results occurred mainly because of two types of events. First, the FV intensity of certain cells was measured above the viable FV intensity range, and subsequent measurements were below this range. Between the two measurements, there were cells whose FV intensity values were quantified within the viable FV intensity. This event may be attributed to a higher *p*-value calculated in TLT cells (when comparing FV intensities in viable and dead cells), as this event occurred more frequently in TLT cells (Figure 7b,d). On the other hand, the exact time at which the cell initiates the cell death pathway is not known. Thus, the cell could be alive at the time of measurement and initiate cell death later during observation.

### 3.4. Cell Viability Prediction Performance of FV-Stained Cells with ISX

After confirming the correlation between the intensity of FV and cell viability, further experiments were needed to observe the performance of MPCVA in predicting cell viability. Since cell seeding density affects the cell cycle, cells were seeded at different cell densities and treated with two concentrations of arsenic (1 mg/L and 5 mg/L) to observe the performance of the assay on cells at different stages of the cell cycle. Analysis was performed using an imaging flow cytometer, which allowed us to measure FV fluorescence intensity as well as cell number in each sample for comparative analysis.

**Figure 8:**
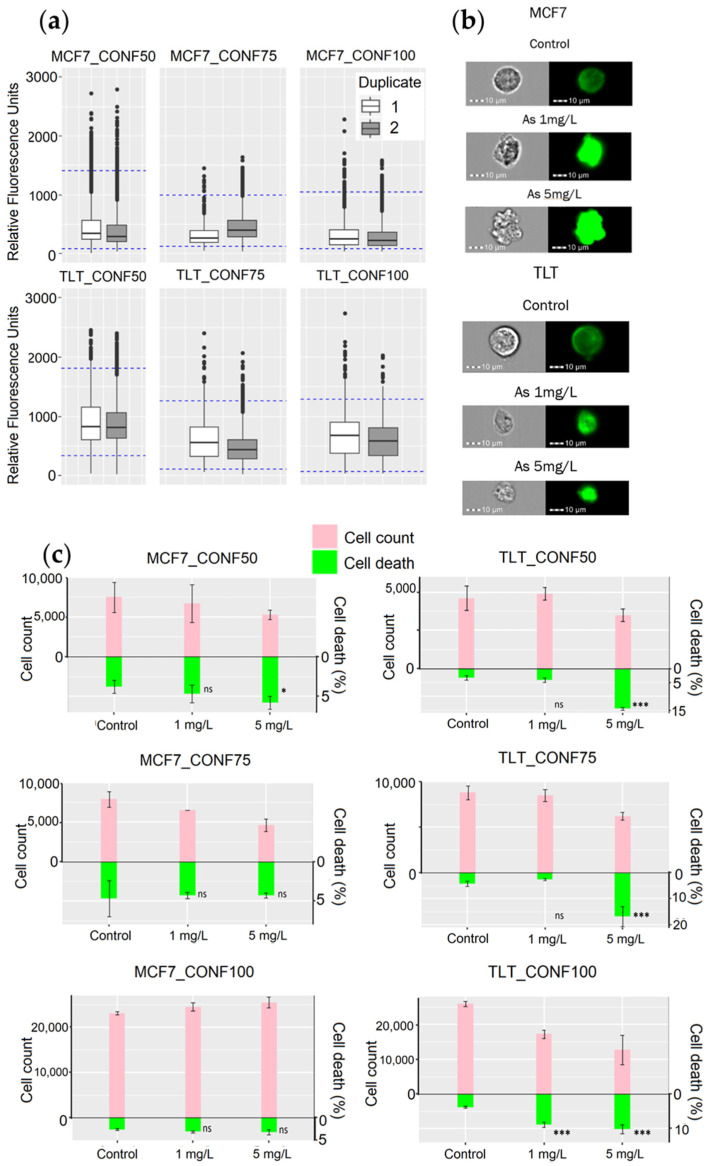
Evaluation of the performance of the MPCVA method for toxicity assessment. (**a**) FV intensity values of the untreated cells (control) measured with the MPCVA method and shown as box plots. The blue dashed lines represent the viable FV intensity ranges of each group of replicates separately. (**b**) The representative images (brightfield and FITC/GFP channel) of MCF7 and TLT cells in MIFC analysis. (**c**) Comparison of the MPCVA method with the cell count method. The top bars (pink) represent the cell number measured by the cell count method. The green bars represent the percentage of cell death measured using the MPCVA method. Samples with a higher percentage of dead cells should have lower cell viabilities after 24 h (cell counting) and vice versa. Cell death % of treated and control (untreated) samples was statistically compared using the Chi-squared method (ns > 0.05, * 0.05, *** 0.001) (labeled below).

The FV intensity range was calculated separately for each cell line and seeding density (Figure 8a). Despite the different seeding densities, the ranges of viable FV intensities were similar in CONF75 and CONF100. In CONF50, where the cell replication rate was higher, the FV intensity was also significantly higher compared to CONF75 and CONF100 in both cell lines. The results are consistent with previous findings where Urrego et al., observed that cells depolarize upon entering G2/M phase [45].

The FV intensity values between duplicates were similar in most groups. Considering that there was a one-hour difference between the measurements of the sample duplicates, it was assumed that the change in membrane potential was permanent for the remaining measured samples.

When comparing the performance of the MPCVA method with the cell count method, we compared the cell death ratio measured using the MPCVA method (Figure 4d) and the cell number measured 24 h later via the cell count method. According to this, the percentage of cell death should be inversely proportional to the cell count of the samples (a higher percentage of cell death is expected in samples with lower cell count and vice versa). In addition, a similar percentage of cell death is expected in samples with similar cell numbers. These considerations were observed when comparing MPCVA cell death predictions and cell numbers in the cell count method.

Nevertheless, cell death was underestimated in MCF7_CONF75 (4.7% CTRL, 4.3% As1, and 4.3% As5) because the cell numbers in the treated samples (6.5 × 10^3^ cells and 4.6 × 10^3^ cells in As 1 and As 5, respectively) were significantly lower than in the untreated samples (7.9 × 10^3^ cells) (Figure 8c, MCF7_CONF75). When we statistically evaluate the results, we find significant differences (*p*-value = 2.2 × 10^−16^) between the duplicates of the untreated samples (Figure 8a, MCF7_CONF75). Extensive cell division may depolarize the cells of the second duplicate (at a much higher ratio than in the first duplicate), resulting in a significant difference in the FV intensity values between the duplicates of the untreated samples in MCF7_CONF75. Although such events are considered rare because the M2 phase of the cell cycle is rapidly completed [11] and the probability of measuring FV intensity at this time is low, it is still important to minimize the occurrence of false-positive events. These differences in FV intensity values, due to cell depolarization, have exceeded the limit of the “viable intensity range” and have led to an underestimation of cell death in the untreated samples. The MPCVA calculations in TLT cells showed an even better correlation and proportionality between the cell death rate and cell count than that observed in MCF7 (Figure 8c).

### 3.5. Comparison with Other Established Methods for Cell Viability Assessment

To further evaluate the suitability of the MPCVA method for determining cell viability, we compared its performance with three commonly used (and well-established) methods for assessing cell viability, namely the PI assay, the MTT assay, and cell number.

**Figure 9:**
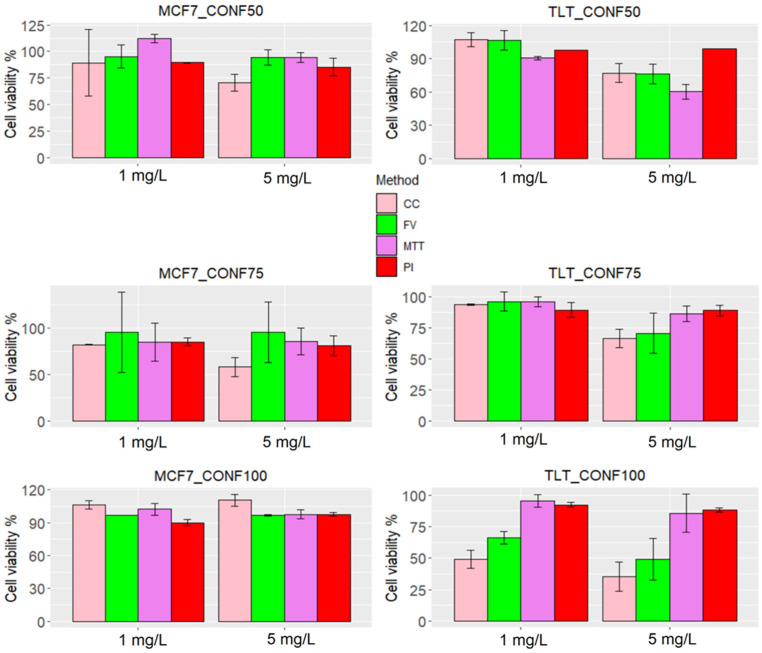
Cell viability of each method in MCF7 and TLT cell lines. The measurement of the treated samples (1 mg/L and 5 mg/L arsenic) was compared with the measurement of the control group and presented as % viability. The viability of each group is shown with different colors: cell number is shown with a pink bar, MPCVA with a green bar, MTT with a purple bar, and PI with a red bar.

The assessment of viability using the MPCVA method was similar to the PI method in samples where viability was high (as measured via the cell count method), (Figure 9). In contrast, the correlation of PI with the MPCVA method was poor in samples with lower viability (measured via the cell count method), mainly because the percentage of cell death estimated using the PI method was already high in the untreated MCF7 samples (e.g., the percentage of cell death was more than 10% in the first three replicates). Because of this high percentage of cell death in the control group, there were no discernible differences between treated and untreated samples (thus, the viability is estimated to be high). The high estimate of cell death in the untreated samples could be a result of extensive cell division, which increases the fluidity of the cell membrane and facilitates the penetration of PI. Inaccuracies in the assessment of cell death using the PI method have also been observed in previous studies [2,29,46].

The prediction of cell death with MTT also showed inconsistencies with MPCVA (and the cell count method). Arsenic impairs the cell cycle (by inducing cell cycle arrest) and the Krebs cycle [46]. Thus, a higher metabolic rate in the untreated cells in TLT CONF 50 should increase formazan production, while cells treated with arsenic should have a lower production rate. Both events may lead to an underestimation of the OD value in the test group, and thus, an overestimation of cell death. (Figure 9). In contrast, the control group in TLT CONF 100 had a very high cell number (more than 25,000 cells), which may lead to an underestimation of formazan production [47]. At the same time, arsenic was more diluted in both treated groups than in the other groups (because of the higher amount of cells), resulting in a lower availability of arsenic to each cell. Consequently, these events may lead to an underestimation of cell death [46]. However, additional tests may be performed to fully understand the reason for the observed results (Figure 9).

### 3.6. MPCVA Method Suitability with Alternative Cell Death Pathways

In the previous section, we evaluated the reliability of our method with standard methods when cell death was induced with arsenic (1 mg/L and 5 mg/L). Our data suggest that the MPCVA method may be as reliable as other standard viability methods, or even more accurate than the PI method, under the conditions described. In this section, we evaluated the suitability (and accuracy) of the MPCVA method when other toxic agents are used to induce cell death.

We added an additional step to the protocol to improve the assay performance for samples with increased cell division (as in Figure 8—MCF7_CONF75), as cell division can lead to changes in membrane potential, resulting in false positives. To identify dividing cells, we added a DNA dye (Vybrant ^TM^) proportional to the DNA content, to the protocol (Appendix A.3). This allowed us to identify cells with higher FV intensities that were dividing, and exclude them from the analysis. In addition, the cell number in each well was recorded and considered for the evaluation of MPCVA viability.

**Figure 10:**
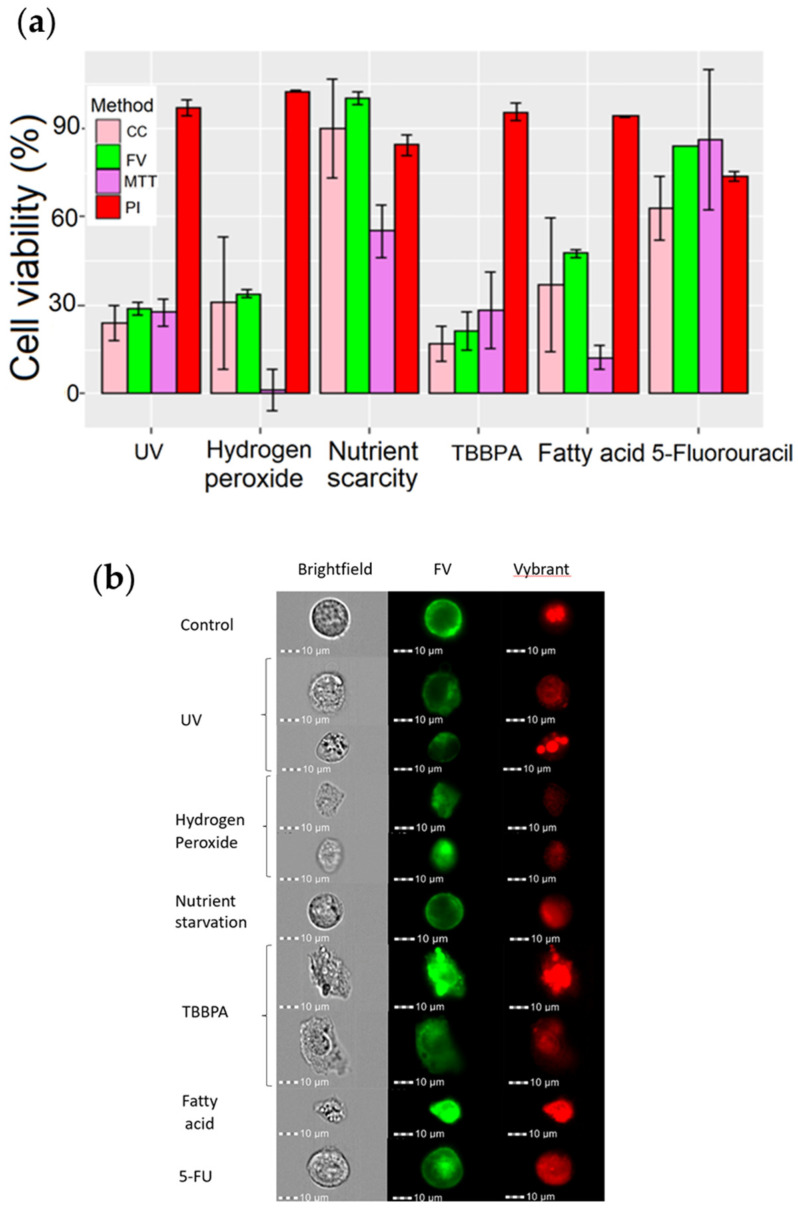
Assessment of cell death in TLT cells after using different toxic substances. TLT cells were treated with different toxic substances to induce programmed cell death. In the first group, cells were irradiated with UV light for 5 min. In the second group, the volume of the medium was reduced from 100 µL to 50 µL for 72 h. In the other groups, 5-fluorouracil, TBBPA, hydrogen peroxide, and fatty acids were added to the cell culture medium at a final concentration of 500 µM, 266 µM, 12.5 mM, and 2% (2/100 µL), respectively. (**a**) Cell viability was assessed for each group and compared to controls. Viability measured via cell count method is shown with a pink bar, MPCVA with a green bar, MTT with a purple bar, and PI with a red bar. (**b**) Representative images of TLT (brightfield, FV—green, Vybrant—red) were acquired for each group during MIFC analysis.

The assessment of cell viability using the MPCVA method yielded similar results to the cell count method, and is consistent with the observations shown in Figure S1. The MPCVA method predicted that cells treated with UV, H_2_O_2_, and TBBPA had the lowest viabilities, whereas starved cells and cells treated with 5 FU had the highest viabilities (Figure 10a). The addition of cell count measurement to the protocol increased the performance of the assay, especially during UV and H_2_O_2_ exposure, where the cell count at the time of the assay performance (24 h post-inoculation) was significantly lower than in the untreated sample. This means that many of the cells treated with UV and H_2_O_2_ were already dead and lysed by the time we performed the assay (because cell death pathways take different lengths of time, mainly 2–48 h). The addition of a DNA stain also increased the accuracy of the assay, and provides valuable information. In this situation, we observed an induction of cell arrest by the chemical 5- FU, which was also observed in previous studies (Figure S2) [48]. We also observed possible DNA condensation in the cells treated with TBBPA (the DNA dye area and intensity are higher than in the control group) or DNA damage in the cells treated with hydrogen peroxide (where there was no DNA dye signal) (Figure 10b). These observations are consistent with observations from previous studies [49]. The viability estimates from the MPCVA and cell count methods were higher, which correlates with the viability observed in Figure S1. The treatments could decrease the metabolic rate in the cells, resulting in a lower reduction of the tetrazolium salt.

Viability estimates using the PI and MPCVA methods were comparable with cells treated with nutrient starvation and 5-fluorouracil, the less cytotoxic treatments. For the remaining samples, the assessment of viability using the PI method differed from the other methods. In the remaining samples, there was a significant reduction in the cell population within the first 24 h after treatment. Thus, most cells were already lysed and the remaining cells were assessed as being viable at the time of viability assessment using the PI.

## 4. Discussion

### 4.1. Overall Applicability of the MPCVA Method

There are many different established methods for determining cell viability. Despite their generally undeniable applicability and ease of use, it must be emphasized that most of these methods rely on the indirect measurement of cell viability (e.g., the measurement of metabolic activity). Consequently, many studies have reported misleading results regarding the accuracy of these methods (e.g., MTT or PI) [2,3,30,50]. Knowing the limitations of each method, it is worthwhile to explore possible new methods to overcome these accuracy problems.

MPCVA could be a good alternative method because it has the advantages of the MTT assay and PI method (easy and fast performance). Since it is based on a direct measurement of cell viability, it should lead to a more accurate assessment of cell death than indirect methods. To prove this, we performed several tests. First, we established a relationship between cell viability and dye intensity (membrane potential) by showing that viable and dead cells have significantly different FV dye intensities. In a series of experiments exposing cells to different physical and chemical toxins, from low to high rates of cell death, we compared the accuracy of the method with standard methods for assessing cell viability (cell count, MTT, and PI). Under the given conditions, the results obtained via the MPCVA method were closest to those obtained via the cell count method. This might be related to the principle of the method, since the MPCVA method is based on the direct determination of cell death, and considers the point of no return. In addition, the MPCVA method is compatible with live cell imaging, which allows for more comprehensive studies of cell viability.

### 4.2. The Potential Use of the MPCVA Method to Determine Cell Death

When analyzing the viability of the cells using the MPCVA method, we noted several trends that varied depending on the experimental conditions. First, we observed two distinguishable trends when analyzing the suitability and accuracy of the MPCVA method with arsenic. To understand these two trends, we combined the results obtained with the fluorescence microscope and the MIFC. With the latter, we analyzed the intensity values of each cell’s FV, its area, and the dye loading (area covered by the fluorophore normalized to the area of the cell). Finally, we linked these results to previous studies found through literature searches [5,6,51,52].

In the TLT, we observed a predominant trend within the dead cells, in which their FV intensities increased significantly and then suddenly decreased. At the same time, we observed that their areas shrank significantly (Figure 8b). These observations are consistent with previous studies of apoptotic cell death [51], in which a drop in cell membrane potential due to potassium leakage (the initial high FV intensity values) was followed by a substantial shrinkage in cell volume. Finally, the formation of apoptotic bodies most likely caused the FV drop in intensity [5,51].

We observed that the trend in which cells exhibited a more uniform decrease in intensity was more frequent in the MCF7 cell line. The majority of dead cells had higher dye loading and a larger cell volume than the putative viable cells (Figure 8b). Higher dye loading and a larger cell volume indicate cell membrane disruption, which further facilitates dye penetration into the cell membrane (Figure 8b). Considering that MCF7 cells have an interrupted apoptotic pathway [53,54], we can assume that this trend may represent a necrotic pathway [6,52].

When we later improved the protocol (using DNA staining dye and cell number determination), we observed additional trends when we tested the method with six different cell death inducers. Nevertheless, further studies are needed to fully understand Vybrant and Fluovolt dynamics in cells treated with the different cell death inducers, and their possible correlation with cell death pathways.

The addition of DNA staining agents allowed us to analyze DNA content and cell cycle. Among the cells treated with 5-fluorouracil, a significant number of cells (23%) were in S phase (Figure S2), indicating that 5-fluorouracil causes cell cycle arrest. This biological response was previously reported by Focacceti et al. [48] and Longley et al. [55].

Most of the cells treated with TBBPA had an irregular shape. At the same time, we found that the DNA content was condensed in many cells and highly scattered in many others (Figure 10b). This observation correlates with apoptotic cell death, in which membrane leakage and chromatin condensation occur at an initial stage, while apoptotic body formation and DNA fragmentation occur at a later stage [5,53,55]. The induction of apoptotic cell death via TBBPA was previously observed by Zhang et al. [39]. However, the enlargement of the cells could also indicate that the cells were undergoing necroptosis.

The DNA content was very low and the cell size was significantly smaller when the cells were cultured with FA (Figure 10b). The addition of FA most likely led to an increase in lipid peroxidation, which eventually resulted in excessive ROS lipid release and DNA damage [49]. We also observed that the addition of FA resulted in the highest FV intensity values that we observed among the toxic substances (Figure 10b). This was expected, since the loss of membrane integrity is a major feature of cell death via ferroptosis [49].

The addition of hydrogen peroxide to the cell medium resulted in polarized FV intensity values, which we observed to be characteristic of an apoptotic pathway. We also observed that the DNA content was low (as there was no signal in the Vybrant channel), which is consistent with the literature (as hydrogen peroxide damages DNA [38]). However, the low DNA content could indicate nuclear degeneration and leakage, which are closely associated with necroptosis [36].

On average, cells exposed to nutrient deprivation had a larger and more scattered DNA staining area than untreated cells (Figure 10b). These observations are consistent with the literature, in which autophagic cells are characterized by the absence of chroma-tin condensation [53]. Most cells had FV intensity values within the FV intensity range, which correlates with the low percentage of cell death observed in this group.

The DNA contents of the cells treated with UV were extremely high in some cells and extremely low in others. Most cells had lost their original shape and had a lower FV intensity than the untreated cells (Figure 10b). This polarity was observed in cells thought to undergo apoptotic cell death [41]. However, considering the cell enlargement, DNA condensation, and large-scale fragmentation, the cells may have followed a parthanatos death pathway [49].

In summary, our assumptions explaining these trends are based in part on the observations of previous studies [5,38,39,41,48,49,56], in which characteristic markers associated with specific cell death pathways were observed. Further studies using cell death markers known to be associated with specific cell death pathways (e.g., apoptosis and caspase, necrosis and RIPK1, etc.) could elucidate the significance of the observed cell death trends.

### 4.3. Summary of the MPCVA Tests

To facilitate the consideration of the MPCVA method in different experimental setups as a method for assessing cell viability, we summarize the main results of our study in the table below (Table 1):

### 4.4. Limitations of the MPCVA Method

The assay is considered universal, but it should be taken into account that neuron type cells have a higher capacity to depolarize than other cell types [11]. A comprehensive analysis should clearly distinguish between transient and permanent membrane depolarization in this cell type.

We did not test the assay with heterogeneous samples, but the different cell types can be separated by morphological features or additional markers (e.g., CD68 antibody for macrophages, or albumin antibody for hepatocytes).

Although the development of such assays could be quite a problematic experimental challenge (high cost, a lot of data to be processed, etc.), the MPCVA method should be further tested by measuring the FV fluorescence values continuously, rather than at 12 h intervals. The values of FV fluorescence intensity between measurements are currently unknown. More frequent measurements could shed further light on the mechanism behind the observed responses in various experiments (e.g., in Figure 6).

The cell has many different mechanisms for repairing membrane damage (membrane repair and fusion, the removal of damaged membranes, or protein-centric repair, to name a few) [57] that could alter the dynamics of dye intensity. Understanding the role of these mechanisms in the cell death pathway should facilitate the correlation between dye intensity and cell viability (and also shed light on the trends we observed). At the same time, the use of MPCVA should also increase our understanding of the mechanisms involved in membrane repair.

To date, there is no evidence to support the use of membrane potential-sensitive dyes as an indicator of cell viability. Therefore, many additional tests may be required before a final validation and the general use of the method [58,59]. Among the possible tests, we consider the latter to be the most important: testing the method with different cell types, using different cell models, generating complete toxicity curves, and testing cytotoxic agents with different toxicodynamic properties. In addition, the method should be tested under the same conditions in different laboratories to allow for comparisons between laboratories. There are many unknowns associated with the method (including the different trends that we observed), leaving room for improvement. During the suitability tests as a cell viability method, we made improvements in the accuracy and applicability of the MPCVA method. We are confident that both elements can be further improved.

## 5. Conclusions

Considering all of the results, we can conclude that the MPCVA method proves to be a suitable assay for determining cell viability, and that there is a relationship between cell membrane potential and cell viability. Under the given conditions, the MPCVA method provided similar results for toxicity assessment to the standardized methods, and has a more promising accuracy, because it directly measures cell viability. It should also allow for more comprehensive studies to assess cell death, as it is compatible with live cell imaging. Therefore, we encourage further studies in alternative settings to further substantiate our preliminary results of the MPCVA method in the context of the assays presented here.

## Figures and Tables

**Table 1:** Summary of the characteristics of the MPCVA method.

Characteristic	Description
**Determination of correlation**	The correlation of cell viability and FV intensity was observed using a fluorescence microscope. After finding a significant correlation, we increased the sample size and added toxic substances to observe the accuracy and universality of the assay using an imaging flow cytometer. In this way, we demonstrated a correlation between membrane potential and cell viability.
**Suitability for different cell types**	The method was tested on epithelial cells and macrophages, and confirmed to be suitable for monitoring cell viability. The method should be further tested with special attention to fibroblasts and neuronal cells, to confirm its suitability for these cell types as well.
**Low toxicity of the FV dye**	We monitored the cells for 80 h and found no signs of toxicity in the control groups (the cells continued to proliferate 80 h after the introduction of the dye). The low toxicity using the improved protocol (with the addition of a DNA dye) should be further tested over 80 h, as the latter dye could affect cell proliferation.
**Monitoring capability**	We were able to monitor cells for 80 h and observe cell replication (unlike MTT and PI, for example). This capability allows for more detailed and continuous observation of cell death. In addition, the results of the fluorescence microscope and the flow cytometer can be compared.
**Applicability independent of cell confluence %**	The confluence of the cells (tested at 50, 75, and 100% confluence) did not affect the performance of the assay.
**High reproducibility of results**	Single viable cells had similar FV intensity values during the 80 h monitoring period. We also observed a similar range of viable FV intensity between samples with different confluence values. This indicates that the method is reproducible.
**Independent of dye loading capacity**	Compensation of dye intensity with dye area effectively prevented degradation of method performance at different dye loadings.
**Applicable for proliferating cells**	We performed the experiment at 50% and 75% confluency (among other confluency values), which means that the cells proliferated. Few false positives were observed in these samples, which could be avoided with the improved protocol (addition of DNA stain).
**Sensitivity to different cell death rates**	We tested the method with different toxic substances at different concentrations to induce different cell death rates (from low to extremely high cell death). The accuracy of the MPCVA method was constant in the different scenarios.
**High accuracy**	The assay has high accuracy in predicting the cell death rates of the samples. The improved assay (using DNA dye and cell number) avoided possible false-positive events related to cell proliferation.
**Flexible**	Considering the experimental setup and the desired outcome of the study, the method can be modified. If the experimental setup is prone to false positives or false negatives, the method can be modulated to reduce these events (by decreasing or increasing the range of viable FV intensity).
**Possible extensions of the protocol with other dyes**	By adding a DNA dye, and by compensating with the cell number of each sample while performing the assay, the method could make even more accurate predictions of cell death events. Thus, the dye FV can be used with other dyes to increase the power of the method.
**Comparable results with other established methods in predicting cell viability**	The improved MPCVA method provided comparable results for cell viability as the cell counting method. In addition, the improved MPCVA method was more reliable in assessing cell viability than the MTT assay and the PI method in certain cases.
**Determination of cell death pathway**	We observed different trends in the results of the method for different toxicants. This may indicate that the assay can detect different cell death pathways.

**Figure A1:**
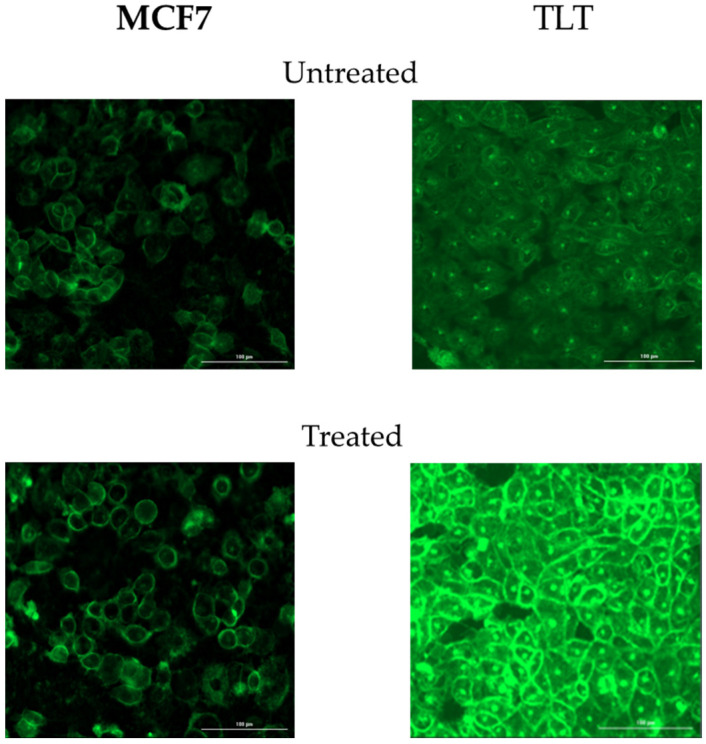
Fluorescence images of treated (12.5 nM peroxide) and untreated MCF7 and TLT cells.

## Supplementary Materials

The following supporting information can be downloaded at: https://www.mdpi.com/article/10.3390/cells11152314/s1. Figure S1: Image of TLT cells (using a ZEISS Axiovert 40 CFL inverted microscope) cultured for 48 h after specific treatment was applied. Figure S2: Frequency of cell cycle phases of each sample.

## Appendix A

In Appendix A, we describe in detail all of the protocols we followed, in the different tests we performed.

### Appendix A.1. Protocol

The detailed protocol for FV staining and fluorescence microscopy:

1. Culture cells in a 96-well plate until the desired confluence is reached.
2. Carefully aspirate media and add 50 µL of the FluoVolt™ Loading Solution. Incubate in the incubator for 30 min.
3. Remove the FluoVolt™ Loading Solution and wash twice with PBS.
4. Add the medium with the target contaminant. Monitor the cells with a fluorescence microscope capable of reading the FITC channel (we did not monitor the cells with the fluorescence microscope with the added DNA stain, but we recommend the addition of the latter).

### Appendix A.2. Protocol

The detailed protocol for FV staining, adjusted for use with flow cytometry:

1. Culture cells in a 96-well plate until the desired confluence is achieved.
2. Carefully aspirate the medium and add the medium containing the target contaminant. Culture the cells for 24 h.
3. Gently aspirate the medium and detach the cells with 100 µL trypsin. After detachment, pipette the cells with the trypsin medium into an Eppendorf. Add 500 µL PBS and centrifuge the Eppendorf at 330 g for 5 min.
4. Carefully aspirate the supernatant. Add 500 µL PBS and centrifuge the Eppendorf at 330 g for 5 min.
5. Gently aspirate the supernatant. Add 50 µL FluoVolt™ Loading Solution. Incubate for 30 min in the incubator.
6. Remove the FluoVolt™ Loading Solution and wash twice with PBS (following the same procedure as in Point 4b).
7. Add 50 µL PBS and analyze the cells in ISX. FluoVolt™ is excited with a 488 nm laser and is visible on channel 2. Duplicates are measured with at least a 1 h difference.

### Appendix A.3. Protocol

The upgraded final protocol for MPCVA, adjusted to be used with flow cytometry:

1. Culture cells in a 96-well plate until the desired confluence is reached.
2. Carefully aspirate media and add the medium with the target contaminant. Culture the cells for 24 h.
3. Carefully aspirate media and detach cells with 100 µL trypsin. After detachment, pipette the cells containing trypsin medium to an Eppendorf. Add 500 µL PBS and centrifuge the Eppendorf for 5 min at 330 g.
4. Carefully aspirate the supernatant. Add 500 µL PBS and centrifuge the Eppendorf for 5 min at 330 g.
5. Carefully aspirate the supernatant. Add 50 µL of the FluoVolt™ Loading Solution. Incubate in the incubator for 30 min.
6. Remove the FluoVolt™ Loading Solution and wash twice with PBS (with the identical procedure as Point 4b).
7. Add 50 µL Vybrant™ Ruby DyeCycle™ stain solution. Incubate in the incubator for 30 min.
8. Analyze the cells in the ISX. FluoVolt™ is excited with a 488 nm laser and is visible at channel 2. Vybrant™ is excited with a 560 nm laser and is visible at channel 11. Duplicates are measured with at least 1 h difference.

## Appendix B

Since the proposed method is a new one and there are many unknowns to consider, the suitability of the method was tested with two additional instruments: a Varioskan spectrophotometer (Thermo Fischer, Waltham, MA, USA) and a Cytation 5 fluorescence microscope (BioTek, Winooski, VT, USA). MCF7 and TLT cells were grown to confluence in 96-well microplates. Once they reached confluence, the cells were stained with FV dye under the same conditions as described in the A1 protocol. Then, 12.5 mM peroxide was added to the treated group and cell medium was added to the untreated group. After 4 h, the microtiter plate was analyzed using both instruments.

When the fluorescence was evaluated using a spectrophotometer, the treated MCF7 cells had 33.3% higher fluorescence than the untreated cells. For TLT cells, the untreated cells had 12.5% higher fluorescence than the untreated cells.

When the fluorescence of the cells was analyzed using a fluorescence microscope, the difference in FV between the treated and untreated cells was clearly visible (Figure A1).

## References

[1] Bunel V., Ouedraogo M., Nguyen A.T., Stévigny C., Duez P. (2014). Methods applied to the in vitro primary toxicology testing of natural products: State of the art, strengths, and limits. Planta Med..

[2] Galluzzi L., Bravo-San Pedro J.M., Vitale I., Aaronson S.A., Abrams J.M., Adam D., Alnemri E.S., Altucci L., Andrews D., Annicchiarico-Petruzzelli M. (2015). Essential versus accessory aspects of cell death: Recommendations of the NCCD 2015. Cell Death Differ..

[3] Wang P., Henning S.M., Heber D. (2010). Limitations of MTT and MTS-based assays for measurement of antiproliferative activity of green tea polyphenols. PLoS ONE.

[4] Jeong H., Tombor B., Albert R., Oltvai Z.N., Barabási A.L. (2000). The large-scale organization of metabolic networks. Nature.

[5] Bortner C.D., Gomez-Angelats M., Cidlowski J.A. (2001). Plasma membrane depolarization without repolarization is an early molecular event in anti-Fas-induced apoptosis. J. Biol. Chem..

[6] Zhang Y., Chen X., Gueydan C., Han J. (2018). Plasma membrane changes during programmed cell deaths. Cell Res..

[7] Styblo M., Del Razo L.M., Vega L., Germolec D.R., LeCluyse E., Hamilton G.A., Reed W., Wang C., Cullen W.R., Thomas D.J. (2000). Comparative toxicity of trivalent and pentavalent inorganic and methylated arsenicals in rat and human cells. Arch. Toxicol..

[8] Sun Y.K., Wang S.J., Zhao Y.Q. (2009). Effect of arsenic pentaoxide on proliferation and apoptosis of human umbilical vein endothelial cell. Zhongguo Yi Xue Ke Xue Yuan Xue Bao Acta Acad. Med. Sin..

[9] Tse W.P., Cheng C.H., Che C.T., Lin Z.X. (2008). Arsenic trioxide, arsenic pentoxide, and arsenic iodide inhibit human keratinocyte proliferation through the induction of apoptosis. J. Pharmacol. Exp. Ther..

[10] Rainieri S., Conlledo N., Langerholc T., Madorran E., Sala M., Barranco A. (2017). Toxic effects of perfluorinated compounds at human cellular level and on a model vertebrate. Food Chem. Toxicol..

[11] Boron W.F., Boulpaep E.L. (2012). Medical Physiology: A Cellular and Molecular Approach.

[12] Wright S.H. (2004). Generation of resting membrane potential. Adv. Physiol. Educ..

[13] Dolenšek J., Špelič D., Klemen M.S., Žalik B., Gosak M., Rupnik M.S., Stožer A. (2015). Membrane Potential and Calcium Dynamics in Beta Cells from Mouse Pancreas Tissue Slices: Theory, Experimentation, and Analysis. Sensors.

[14] Bedut S., Seminatore-Nole C., Lamamy V., Caignard S., Boutin J.A., Nosjean O., Stephan J.P., Coge F. (2016). High-throughput drug profiling with voltage- and calcium-sensitive fluorescent probes in human iPSC-derived cardiomyocytes. Am. J. Physiol. Heart Circ. Physiol..

[15] Miller E.W., Lin J.Y., Frady E.P., Steinbach P.A., Kristan W.B., Tsien R.Y. (2012). Optically monitoring voltage in neurons by photo-induced electron transfer through molecular wires. Proc. Natl. Acad. Sci. USA.

[16] Woodford C.R., Frady E.P., Smith R.S., Morey B., Canzi G., Palida S.F., Araneda R.C., Kristan J.W.B., Kubiak C.P., Miller E.W. (2015). Improved PeT molecules for optically sensing voltage in neurons. J. Am. Chem. Soc..

[17] Veech R.L., Kashiwaya Y., King M.T. (1995). The resting membrane potential of cells are measures of electrical work, not of ionic currents. Integr. Physiol. Behav. Sci..

[18] Núñez R., Sancho-Martínez S.M., Novoa J.M.L., López-Hernández F.J. (2010). Apoptotic volume decrease as a geometric determinant for cell dismantling into apoptotic bodies. Cell Death Differ..

[19] Park J., Werley C.A., Venkatachalam V., Kralj J.M., Dib-Hajj S.D., Waxman S.G., Cohen A.E. (2013). Screening fluorescent voltage indicators with spontaneously spiking HEK cells. PLoS ONE.

[20] Rienecker K.D.A., Poston R.G., Saha R.N. (2020). Merits and Limitations of Studying Neuronal Depolarization-Dependent Processes Using Elevated External Potassium. ASN Neuro.

[21] Koley D., Bard A.J. (2010). Triton X-100 concentration effects on membrane permeability of a single HeLa cell by scanning electrochemical microscopy (SECM). Proc. Natl. Acad. Sci. USA.

[22] Demuynck R., Efimova I., Lin A., Declercq H., Krysko D.V. (2020). A 3D Cell Death Assay to Quantitatively Determine Ferroptosis in Spheroids. Cells.

[23] Messam C.A., Pittman R.N. (1998). Asynchrony and commitment to die during apoptosis. Exp. Cell Res..

[24] Aragane Y., Kulms D., Metze D., Wilkes G., Pöppelmann B., Luger T.A., Schwarz T. (1998). Ultraviolet light induces apoptosis via direct activation of CD95 (Fas/APO-1) independently of its ligand CD95L. J. Cell Biol..

[25] Rehemtulla A., Hamilton C.A., Chinnaiyan A.M., Dixit V.M. (1997). Ultraviolet radiation-induced apoptosis is mediated by activation of CD-95 (Fas/APO-1). J. Biol. Chem..

[26] Rosette C., Karin M. (1996). Ultraviolet light and osmotic stress: Activation of the JNK cascade through multiple growth factor and cytokine receptors. Science.

[27] Elmore S. (2007). Apoptosis: A Review of Programmed Cell Death. Toxicol. Pathol..

[28] Stožer A., Dolenšek J., Rupnik M.S. (2013). Glucose-stimulated calcium dynamics in islets of Langerhans in acute mouse pancreas tissue slices. PLoS ONE.

[29] Wiepz G.J., Edwin F., Patel T., Bertics P.J. (2006). Methods for determining the proliferation of cells in response to EGFR ligands. Methods Mol. Biol..

[30] Rieger A.M., Nelson K.L., Konowalchuk J.D., Barreda D.R. (2011). Modified annexin V/propidium iodide apoptosis assay for accurate assessment of cell death. J. Vis. Exp..

[31] van Meerloo J., Kaspers G.J., Cloos J. (2011). Cell sensitivity assays: The MTT assay. Methods Mol. Biol..

[32] Zuba-Surma E.K., Kucia M., Abdel-Latif A., Lillard J.W., Ratajczak M.Z. (2007). The ImageStream System: A key step to a new era in imaging. Folia Histochem. Cytobiol.

[33] Li J., Cao F., Yin H.L., Huang Z.J., Lin Z.T., Mao N., Sun B., Wang G. (2020). Ferroptosis: Past, present and future. Cell Death Dis..

[34] Vanlangenakker N., Vanden Berghe T., Vandenabeele P. (2012). Many stimuli pull the necrotic trigger, an overview. Cell Death Differ..

[35] Fatokun A.A., Dawson V.L., Dawson T.M. (2014). Parthanatos: Mitochondrial-linked mechanisms and therapeutic opportunities. Br. J. Pharmacol..

[36] Robinson N., Ganesan R., Hegedűs C., Kovács K., Kufer T.A., Virág L. (2019). Programmed necrotic cell death of macrophages: Focus on pyroptosis, necroptosis, and parthanatos. Redox Biol..

[37] Cho K.-H., Choi S.-M., Kim B.-C., Lee S., Park M.-S., Kim M.-K., Kim J.-K. (2004). 5-fluorouracil-induced oligodendrocyte death and inhibitory effect of cycloheximide, Trolox, and Z-VAD-FMK in murine cortical culture. Cancer.

[38] Xiang J., Wan C., Guo R., Guo D. (2016). Is Hydrogen Peroxide a Suitable Apoptosis Inducer for All Cell Types?. BioMed Res. Int..

[39] Zhang Y., Wang X., Chen C., An J., Shang Y., Li H., Xia H., Yu J., Wang C., Liu Y. (2019). Regulation of TBBPA-induced oxidative stress on mitochondrial apoptosis in L02 cells through the Nrf2 signaling pathway. Chemosphere.

[40] Noguchi M., Hirata N., Tanaka T., Suizu F., Nakajima H., Chiorini J.A. (2020). Autophagy as a modulator of cell death machinery. Cell Death Dis..

[41] Salucci S., Burattini S., Battistelli M., Baldassarri V., Maltarello M.C., Falcieri E. (2012). Ultraviolet B (UVB) irradiation-induced apoptosis in various cell lineages in vitro. Int. J. Mol. Sci..

[42] McCann F.V., Cole J.J., Guyre P.M., Russell J.A. (1983). Action potentials in macrophages derived from human monocytes. Science.

[43] Berzingi S., Newman M., Yu H.-G. (2016). Altering bioelectricity on inhibition of human breast cancer cells. Cancer Cell Int..

[44] Bertrand C.A., Laboisse C., Hopfer U., Bridges R.J., Frizzell R.A. (2006). Methods for detecting internalized, FM 1-43 stained particles in epithelial cells and monolayers. Biophys. J..

[45] Urrego D., Tomczak A.P., Zahed F., Stühmer W., Pardo L.A. (2014). Potassium channels in cell cycle and cell proliferation. Philos. Trans. R. Soc. Lond. B Biol. Sci..

[46] Medda N., De S.K., Maiti S. (2021). Different mechanisms of arsenic related signaling in cellular proliferation, apoptosis and neo-plastic transformation. Ecotoxicol. Environ. Saf..

[47] Ghasemi M., Turnbull T., Sebastian S., Kempson I. (2021). The MTT Assay: Utility, Limitations, Pitfalls, and Interpretation in Bulk and Single-Cell Analysis. Int. J. Mol. Sci..

[48] Focaccetti C., Bruno A., Magnani E., Bartolini D., Principi E., Dallaglio K., Bucci E.O., Finzi G., Sessa F., Noonan D. (2015). Effects of 5-fluorouracil on morphology, cell cycle, proliferation, apoptosis, autophagy and ROS production in endothelial cells and cardiomyocytes. PLoS ONE.

[49] Yan G., Elbadawi M., Efferth T. (2020). Multiple cell death modalities and their key features (Review). World Acad. Sci. J..

[50] Qi R., Shen M., Cao X., Guo R., Tian X., Yu J., Shi X. (2011). Exploring the dark side of MTT viability assay of cells cultured onto electrospun PLGA-based composite nanofibrous scaffolding materials. Analyst.

[51] Bortner C.D., Cidlowski J.A. (2002). Apoptotic volume decrease and the incredible shrinking cell. Cell Death Differ..

[52] Galluzzi L., Vitale I., Aaronson S.A., Abrams J.M., Adam D., Agostinis P., Alnemri E.S., Altucci L., Amelio I., Andrews D.W. (2018). Molecular mechanisms of cell death: Recommendations of the Nomenclature Committee on Cell Death 2018. Cell Death Differ..

[53] Oh S., Xiaofer E., Ni D., Pirooz S.D., Lee J.-Y., Lee D., Zhao Z., Lee S., Lee H., Ku B. (2011). Downregulation of autophagy by Bcl-2 promotes MCF7 breast cancer cell growth independent of its inhibition of apoptosis. Cell Death Differ..

[54] Fani S., Dehghan F., Karimian H., Mun Lo K., Ebrahimi Nigjeh S., Swee Keong Y., Soori R., May Chow K., Kamalidehghan B., Mohd Ali H. (2016). Monobenzyltin Complex C1 Induces Apoptosis in MCF-7 Breast Cancer Cells through the Intrinsic Signaling Pathway and through the Targeting of MCF-7-Derived Breast Cancer Stem Cells via the Wnt/β-Catenin Signaling Pathway. PLoS ONE.

[55] Longley D.B., Harkin D.P., Johnston P.G. (2003). 5-fluorouracil: Mechanisms of action and clinical strategies. Nat. Rev. Cancer.

[56] Bhuyan A.K., Varshney A., Mathew M.K. (2001). Resting membrane potential as a marker of apoptosis: Studies on Xenopus oocytes microinjected with cytochrome c. Cell Death Differ..

[57] Dias C., Nylandsted J. (2021). Plasma membrane integrity in health and disease: Significance and therapeutic potential. Cell Discov..

[58] Leist M., Efremova L., Karreman C. (2010). Food for thought considerations and guidelines for basic test method descriptions in toxicology. Altex.

[59] Krebs A., Waldmann T., Wilks M.F., Van Vugt-Lussenburg B.M.A., Van Der Burg B., Terron A., Steger-Hartmann T., Ruegg J., Rovida C., Pedersen E. (2019). Template for the description of cell-based toxicological test methods to allow evaluation and regulatory use of the data. Altex.

