# Supplementary material for: A Promising Method for the Determination of Cell Viability: The Membrane Potential Cell Viability Assay
Source: Cells. 2022 Jul 27;11(15):2314. doi: 10.3390/cells11152314 (PMC9367465; doi:10.3390/cells11152314)
Supplement: Supplementary file 1 [file cells-11-02314-s001.zip › cells-1829476-supplementary.pdf]

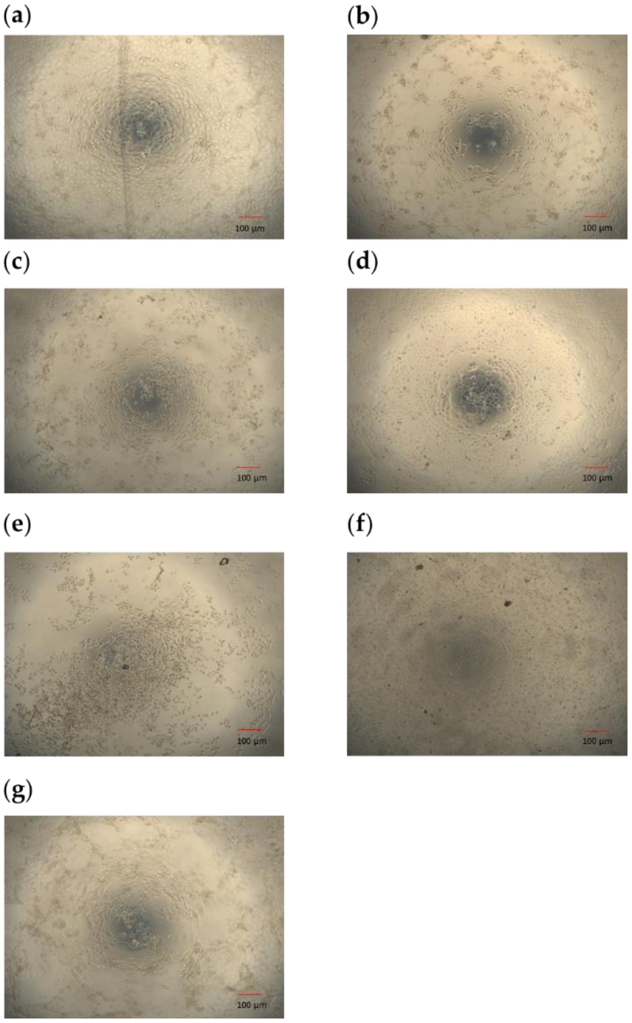

Figure S1. Image of TLT cells (using a ZEISS Axiovert 40 CFL inverted microscope) cultured for 48 h after specific treatment was applied. (**a**) Untreated TLT cells retained the monolayer. (**b**) UV irradiation for 5 min had a visible cytotoxic effect. (**c**) Cells treated with 12.5 mM Hydrogen Peroxide (most cells died and were lysed). (**d**) Nutrient deprivation affected the cell monolayer. (**e**) Cells treated with 266 µM TBBPA experienced a significant reduction in the cell population. (**f**) The addition of Fatty Acid 2% (2/100 µL) to cells resulted in a significant reduction in cell size. (**g**) Cells cultured with 500 µM of5- FU did not retain monolayer.

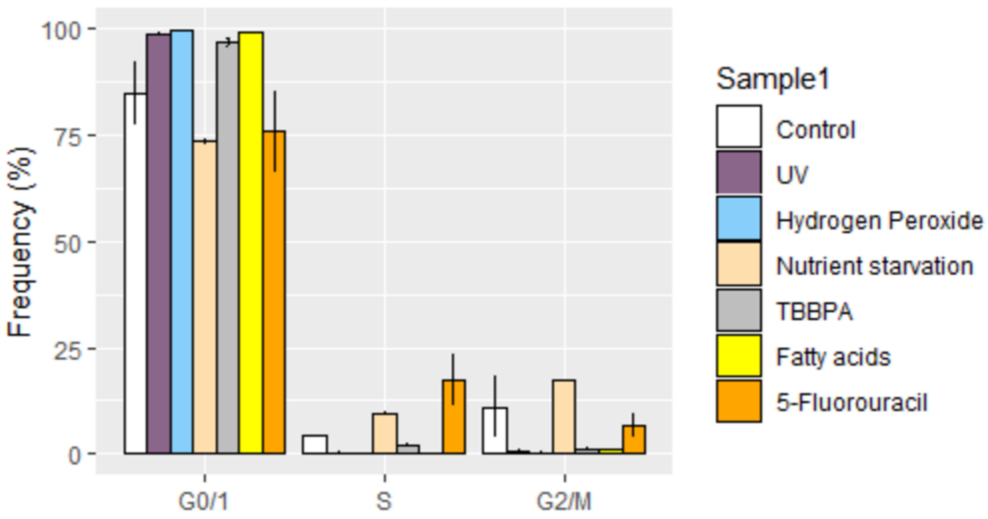

Figure S2. Frequency of cell cycle phases of each sample. The cell cycle phase is shown on the *x*-axis and is restricted to G0/1, S, and G2/M. In each phase, the different samples are represented by bar graphs with different colors.
